# Dietary Regulation of Keap1/Nrf2/ARE Pathway: Focus on Plant-Derived Compounds and Trace Minerals

**DOI:** 10.3390/nu6093777

**Published:** 2014-09-19

**Authors:** Amanda L. Stefanson, Marica Bakovic

**Affiliations:** Department of Human Health and Nutritional Sciences, University of Guelph, 50 Stone Road E, Guelph, Ontario N1G 2W1, Canada; E-Mail: astefans@uoguelph.ca

**Keywords:** Nrf2, NFκB, Keap1, oxidative stress, glutathione, antioxidant enzymes, phase 2 enzymes, nutrient: gene interactions, phytochemicals, trace minerals, zinc, selenium

## Abstract

It has become increasingly evident that chronic inflammation underpins the development of many chronic diseases including cancer, cardiovascular disease and type 2 diabetes. Oxidative stress is inherently a biochemical dysregulation of the redox status of the intracellular environment, which under homeostatic conditions is a reducing environment, whereas inflammation is the biological response to oxidative stress in that the cell initiates the production of proteins, enzymes, and other compounds to restore homeostasis. At the center of the day-to-day biological response to oxidative stress is the Keap1/Nrf2/ARE pathway, which regulates the transcription of many antioxidant genes that preserve cellular homeostasis and detoxification genes that process and eliminate carcinogens and toxins before they can cause damage. The Keap1/Nrf2/ARE pathway plays a major role in health resilience and can be made more robust and responsive by certain dietary factors. Transient activation of Nrf2 by dietary electrophilic phytochemicals can upregulate antioxidant and chemopreventive enzymes in the absence of actual oxidative stress inducers. Priming the Keap1/Nrf2/ARE pathway by upregulating these enzymes prior to oxidative stress or xenobiotic encounter increases cellular fitness to respond more robustly to oxidative assaults without activating more intense inflammatory NFκB-mediated responses.

## 1. Introduction

It has become increasingly evident that chronic inflammation underpins the development of many chronic diseases including cancer, cardiovascular disease, and type 2 diabetes [1]. Reactive oxygen species (ROS) are generated by normal energy metabolism and function as important cell signaling molecules. In homeostatic conditions, intracellular ROS are maintained at appropriate concentrations to fulfill these functions and any excesses are buffered by various antioxidant enzymes and molecules. If ROS production increases beyond the threshold of this buffering capacity, these reactive species trigger uncontrolled reactions with non-target intracellular compounds, oxidizing nucleic acids, proteins, membrane, and other lipids. Normal intracellular oxidative status is reducing. As the number of oxidized compounds rises, the intracellular redox state of the cell begins to shift away from its normal reducing environment and oxidative stress ensues.

Oxidative stress is inherently a biochemical dysregulation of the redox status of the intracellular environment, which under homeostatic conditions is a reducing environment, whereas inflammation is the biological response to oxidative stress in that the cell initiates the production of proteins, enzymes and other compounds to restore homeostasis. At the center of the day-to-day biological response to oxidative stress is the Keap1/Nrf2/ARE pathway, which regulates the transcription of many antioxidant genes that preserve cellular homeostasis and detoxification genes that process and eliminate carcinogens and toxins before they can cause damage. Nrf2^−/−^ mice are more sensitive to inflammatory disease [2] and carcinogenesis [3]. This pathway plays a major role in health resilience and can be made more robust and responsive by certain dietary factors such as phytochemicals and trace minerals.

## 2. Role of Nrf2 as Antioxidant and Chemoprotective Regulator

Encoded by the *NFE2L2* gene, Nrf2 (nuclear factor [erythroid-derived 2]-like 2), is a transcription factor in the basic leucine zipper (bZIP) superfamily with a Cap‘n’Collar (CNC) structure. Nrf2 heterodimerizes with small Maf proteins in the nucleus; this complex can then bind its cognate response element, the antioxidant response element (ARE), which upregulates the transcription of ARE-responsive genes [4,5] (ARE sequence reviewed here [6]). Synthesis of many proteins with well-characterized roles in cellular antioxidant and detoxification pathways is inducible by Nrf2 via one or more AREs in the promoter regions of the corresponding genes; key proteins that can be affected by dietary factors will be briefly summarized.

### 2.1. Antioxidant

Many oxidative cell metabolites have important roles in cell signaling. Present in excess, they can cause uncontrolled oxidative damage to other intracellular compounds (DNA, proteins, plasma membrane, and other lipids). Antioxidants by definition can stably maintain more than one redox state and can therefore participate in electron transfer, including metals that can have two or more redox states (Cu^1+/2+^, Fe^2+/3+/4+^). They generally do not exist freely in the cytosol, but are rather stored and transported by specialized proteins, or are incorporated into enzymes where their redox cycling potential is controlled. The sulfur (S) atom has six potential redox states under biological/physiological conditions [7].

#### 2.1.1. ARE-Responsive Enzymes Associated with Glutathione

Glutathione (GSH) is a three amino acid peptide thiol that is arguably the most abundant intracellular antioxidant protein, present in the cytosol at approximately 5 mM concentrations [8]. Nrf2 is key to regulating GSH levels by upregulating GSH synthetic and regenerative enzymes, as well as enzymes using GSH as a cofactor. Glutamate cysteine ligase (GCL) catalyzes the rate-limiting step in GSH synthesis. It is composed of two subunits, GCLC and GCLM. The GCLC subunit alone can catalyze the reaction that produces γ-glutamyl cysteine from l-glutamate and l-cysteine, but the efficiency is increased when bound to the GCLM subunit. Both GCLC and GCLM are upregulated by Nrf2 [8]. Glutathione synthetase (GSS) condenses γ-glutamyl cysteine and glycine, forming GSH [8]. GSH is a non-specific cytoplasmic reducant that is oxidized to the glutathione disulfide form (GSSG). Under homeostatic conditions the GSH:GSSG ratio is approximately 100:1 [9]. GSSG is regenerated to two GSH molecules by the NADPH cofactor of glutathione reductase (GSR), another Nrf2-responsive gene. Glutathione peroxidase (GPx) catalyzes the reduction of hydrogen peroxide to water and lipid peroxides to their corresponding alcohols. The active site of GPx is composed of 2 GSH where selenium is substituted for sulfur in the thiol of one GSH unit. Glutathione-*S*-transferase (GST) is a phase II detoxification enzyme discussed later.

#### 2.1.2. Other ARE-Responsive Antioxidant Enzymes

Superoxide dismutase (SOD) catalyzes a disproportionation reaction transforming highly reactive superoxide (O_2_^‑^**)** into stabilized dioxygen (O_2_) and hydrogen peroxide (H_2_O_2_), which can be further reduced by GPx. SOD is a metalloenzyme (Zn,Cu-SOD) stabilized by zinc using copper as the redox agent in the active site (manganese is the redox agent in mitochondrial SOD) [10]. NAD(P)H: quinone oxidoreductase-1 (NQO-1) is an inducible enzyme, encoded by NQO1 gene. It is of particular importance in that it can fully reduce quinones to hydroquinones by employing a two-electron transfer, thus avoiding the production of free radical oxygen intermediates [11]. Heme oxygenase-1 (HO-1), encoded by the *HMOX1* gene, is an inducible enzyme that catalyzes the freeing of heme-bound Fe to form biliverdin. HO-1 is highly expressed in the spleen, the site of erythrocyte recycling. Biliverdin can then be reduced by biliverdin reductase to bilirubin, releasing carbon monoxide (CO) to anti-inflammatory effect. Catalase is a highly efficient enzyme that reduces hydrogen peroxide to water and oxygen using Fe in the catalytic site [10,12]. Thioredoxin (Trx) is a protein disulfide reductase that is itself reduced by thioredoxin reductase (TrxR). TrxR is a small, 12 kDa, selenoenzyme that regenerates oxidized Trx to its reduced form in a NADPH-dependent fashion [13].

#### 2.1.3. Other Proteins Relevant to Redox, not Necessarily ARE-Responsive

Metallothioneins (MT) are a family of small (6–7 kDa), cysteine-rich metal storage an transport proteins that bind up seven zinc ions, holding up to 15% of total intracellular zinc [14,15]. At minimum, there are seventeen MT isoforms in the human genome [14]. Although MTs contain an ARE, they are regulated more by the Nrf1 [16] than the Nrf2 isoform [17]. There is debate whether MTs participate in the transfer of free electrons thus playing a role as direct antioxidants; nonetheless, they play an important role in redox biology and it is clear that their metal chelating capacity is regulated by intracellular oxidation status [18,19,20,21]. Different MT isoforms have differential redox potentials and can buffer intracellular free zinc across all physiological zinc concentrations [15,22,23]. Under oxidized conditions and particularly in the presence of Se, MT is a zinc donor; redox activated ligands displace zinc in the zinc/thiolate clusters of MT. Under reduced conditions and after *de novo* synthesis, MT is a zinc acceptor [24,25,26]. Thionein, the MT apoprotein, quickly reacts with zinc ions holding inhibitory positions on other proteins, thus activating them; it can also inhibit transcription factors containing zinc-finger motifs by removing the zinc ion which dramatically alters the protein structure and suppressing DNA binding [26]. Intracellular MT concentration parallels that of free zinc [21].

### 2.2. Detoxification and Neutralization of Carcinogens

Xenobiotics are potentially genotoxic foreign compounds that enter the body at sites of interface with the environment (skin, lung, GI tract, mucous membranes). Hydrophilic xenobiotic molecules are generally excluded entry since they neither diffuse across the plasma membrane, nor are imported by membrane transporters. Biologically relevant xenobiotic molecules tend to be inert, lipophilic compounds that can pass undetected through lipid plasma membranes and evade interaction with transporters due to their hydrophobicity. Once inside cells, they can interfere with cell function in various ways. Detoxification modifies and removes xenobiotic compounds before they can cause damage [27]. The gastrointestinal tract bears the greatest exposure and is the site of first pass detoxification [28], but the liver is the primary organ of detoxification.

Detoxification proceeds in three phases. Enzymes of the cytochrome P450 superfamily are oxidative enzymes that carry out phase 1 by introducing an active site into otherwise inert xenobiotics, creating a highly reactive intermediate [29]. Phase 2 enzymes exploit the newly created active site to conjugate a functional group that solubilizes the xenobiotic, enabling excretion [30]. An imbalance between phase 1 and phase 2 detoxification systems, where there are insufficient phase 2 enzymes to transform the reactive intermediates produced by CYP450 enzymes (P1 > P2), results in increasing oxidative stress, inflammation as well as protein and DNA damage via adduct formation [10]. Multifunctional enhancers of this system upregulate the transcription of both enzyme types, but they are mostly differentially regulated with many phase 2 enzymes falling under the regulation of Nrf2 [30].

#### ARE-Responsive Phase 2 Enzymes

Generally, phase 2 enzymes quench the phase 1-created reactive site by conjugating a functional group that solubilizes the xenobiotic, rendering it excretable [30]. Glutathione-*S*-transferase (GST) is an inducible enzyme that catalyzes the *S*-glutathionylation of xenobiotic compounds. Other electrophilic compounds can replace the newly conjugated R-SG group, but, if not, excretion of these products represents a loss of cellular GSH. UDP-glucuronosyltransferase (UGT) conjugates a glucuronic acid moiety. NQO1 is also sometimes considered a phase 2 enzyme. Additionally, phase 3 (also known as multi-drug resistant) proteins are ARE-responsive; they are a family of membrane transporters that remove processed toxins which become destined for excretion [30].

### 2.3. Synthesis and Regeneration of NADPH

In addition to direct upregulation of ARE-responsive genes, Nrf2 also supports antioxidant and detoxification pathways by increasing the synthesis and regeneration of nicotinamide adenine dinucleotide phosphate (NADPH), a niacin-derived reducing agent. NADPH is a direct antioxidant and is used as an enzyme cofactor in many redox reactions such as the reduction of GSH by GR [31,32]. In normal human breast epithelial cell lines, Nrf2 activation by both Keap1 knockdown (siRNA) and sulforaphane treatment increased the transcription and protein levels of glucose-6-phosphate 1-dehydrogenase (G6PD) and phosphogluconate dehydrogenase (PGD), which are the enzymes in the oxidative arm of the pentose phosphate pathway that regenerate NADPH from NADP^+^ [33]. The A549 cell line (human lung epithelial adenocarcinoma) has a somatic mutation in *KEAP1* resulting in constitutively active Nrf2. Silencing RNA Nrf2 knockdown reduced G6PD and PGD expression in this cell line; additionally, genes involved in NADPH synthesis, malic enzyme 1 (ME1), and isocitrate dehydrogenase 1 (IDH1), were also decreased. ChIP analysis revealed that Nrf2 directly upregulates G6PD, PGD, ME1, and IDH1 via ARE-binding [34]. Similar results of pentose phosphate pathway genes were also observed in a “gene-dose response” study and also demonstrated a corresponding increase of hepatic NADPH relative to Nrf2 stability in Nrf2 null, wild type, Keap1 knockdown and Keap1 knockout mice [35].

ARE-responsive enzymes are key to promoting cell survival by regulating the intracellular redox status protecting against oxidative damage to cell components, and the detoxification processes that protect against protein and DNA adduction that can impair protein function and lead to mutagenesis. Overwhelming these protective systems allows cellular damage to accumulate, representing the first stage of deviation from normal homeostasis that can eventually reinforce the development of many chronic inflammatory diseases. Optimal function of the Keap1/Nrf2/ARE pathway may help to slow or prevent chronic disease progression.

## 3. Regulation of Nrf2

### 3.1. Keap1

Under normal physiological conditions, most Nrf2 is sequestered in the cytosol by its actin-bound inhibitor protein, Kelch-like ECH-associated protein-1 (Keap1) [36], a zinc metalloprotein [37,38] that is localized near the plasma membrane [39]. Keap1 is a 624 amino acid, cysteine rich, homodimeric zinc-finger protein that functions as an adapter for Cul3-Rbx E3 ubiquitin ligase complex [40]. The Cul3-Rbx E3-mediated ubiquination of Nrf2 facilitated by Keap1 ensures constant proteasomal degradation of Nrf2 and inhibits ARE activation. As a result, Nrf2 has a half-life of approximately 20 min under basal conditions [41].

**Figure 1:**
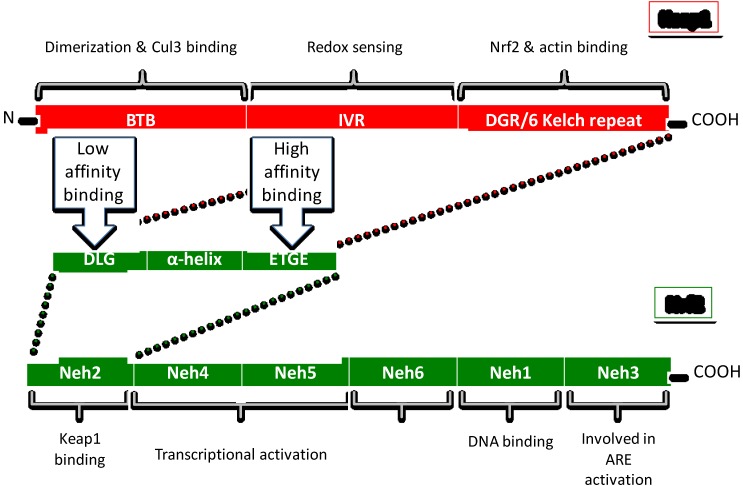
Structure of Keap1 and Nrf2 protein.

Keap1 has three functional domains (Figure 1). Adjacent to the *N*-terminal region, the BTB (bric-a-brac, tramtrac, broad complex) domain is the site of dimerization of the two Keap1 subunits, as well as the binding site of the Cul3-Rbx E3 complex [42]. Keap1 has at least 25 reactive thiols (Cys-SH), most of which are found in the IVR (intervening linker region) redox sensing domain [40,43]. The DGR (double glycine repeat/6 Kelch repeat) domain is the binding site for both actin [36] and Nrf2 [44]. Keap1 has an Nrf2 binding site on each dimer subunit. These two binding sites form the basis of the “latch and hinge” theory of Nrf2 activation [44,45] (discussed later).

#### Redox Sensing Mechanisms of Keap1

There are certain characteristics of Keap1 that allow it to be a highly sensitive oxidation sensor of exogenous electrophiles and oxidation at the plasma membrane. Cytosolic GSH has a greater reaction potential than protein-bound thiol groups to electrophiles. However, actin-bound Keap1 is localized near the plasma membrane and is more likely to have the first encounter with exogenous electrophiles and the products of lipid peroxidation [39,46]. Additionally, different Keap1 thiol groups have different redox potentials. Four of the most reactive cysteine residues on murine Keap1 are located in the IVR (C257, C273, C288, C297) [43]. The commonality across these residues is their position adjacent to basic amino acid residues, which lowers their pKa and contributes to their greater reactivity [43]. C613 in the *C*-terminal domain has a pKa in the range of the four most reactive cysteine residues, although there are currently no clear observations of its redox activity [43,47]. Additionally, Forman *et al*. [46] have proposed that the sulfur atom of a zinc-bound cysteine can acquire a partial negative charge [46]. The exact binding site of zinc is not clear, however Keap1 discharges zinc ions concomitant with Nrf2 release; loss of structural zinc ions may result in the conformational change that fully releases Nrf2 [38] and contribute to intracellular zinc flux. In an examination of the highly conserved role of zinc in redox biology, it has been suggested that binding of free zinc stabilizes and protects critical sulfhydryl groups [48,49]. Particular conditions acting on specific cysteine residues can increase their redox sensing capacity.

Disulfide bridges formed between C273 and C288 are preferential and may be required for Nrf2 activation [21,47,50]. Modification of C273 or C288 alone or in combination abolishes the ability of Keap1 to repress Nrf2 [12]. Not all electrophiles interact with Keap1 in the same way. For instance, both tertbutylhydroquinone (tBHQ) and *N*-iodoacetyl-*N*-biotinylhexylenediamine (IAB) increased HO-1 to a similar degree, but bound Keap1 in different regions [51]. Unlike some other Nrf2 activators, C151 in the BTB domain is required for sulforaphane [12], and falcarindiol induction [52]. However, C151 appears to be the most reactive residue in human Keap1, at least in response to alkylating compounds with a Michael acceptor moiety. Luo *et al.* [53] investigated the reactivity of three different electrophiles and found that while they each had a different binding pattern with Keap1, the alkylation of C151 was always present [53]. Top reactive thiols on human Keap1 were C151 and C266 for isoliquiritigenin, C151, C319, and C613 for xanthohumol, C151, C257, and C368 for 10-shogaol [53], and for iodoacetyl-*N*-biotinyl hexylene diamine they were C151, C288, and C297 [54]. While there is some overlap between the key binding sites on murine and human Keap1, they are not identical.

### 3.2. Nrf2

Nrf2 is a 605 amino acid transcription factor composed of six domains (Neh1-6) (Figure 1). The *N*-terminal Neh2 domain is the binding site for inhibitory protein Keap1 [41]. The Neh4 and Neh5 domains are the transactivation regions that bind other transcription modifiers. The Neh1 domain is the DNA binding region which binds small Maf proteins required for ARE activation [55]. Neh3, adjacent to the *C*-terminal region is involved in ARE activation. Neh2 binds the Keap1 homodimer in two places. It has a high affinity binding site with an ETGE motif and a low affinity site with a DLG motif separated by an alpha helix zone and form the basis of the “latch and hinge” theory of Nrf2 activation [56,57]. It is theorized that the high affinity ETGE motif is the main Keap1 binding site, whereas the low affinity DTG binding site serves as the “latch” that pulls Nrf2 into association with Cul3-Rbx E3 ligase, ensuring the proteasomal degradation of Nrf2 [58]. Consequently, disruption of the low affinity site abrogates Nrf2 degradation but does not release it, making Nrf2 release a two-step process. This insinuates more than one possible route of nuclear accumulation of Nrf2. Electrophilic interaction with Keap1 can disrupt the low affinity/weak DLG “latch” which allows Nrf2 to rotate on a “hinge” that removes it from association with Cul3-Rlx E3 ligase complex and reducing Nrf2 ubiquination. Therefore the pool of Keap1 remains locked into association with existing Nrf2 via the high affinity ETGE site, allowing more *de novo* Nrf2 to escape Keap1 capture and proceed directly to the nucleus [59]. Additionally, further conformational change to Keap1 can release Nrf2, which further contributes to nuclear accumulation. Keap1 is primarily a cytosolic protein with small amounts in the ER and nucleus [60]. Although Keap1 does not co-locate to the nucleus [60], there is current debate whether nuclear Keap1 plays a role in the degradation of nuclear Nrf2 as well.

The presence of both ETGE and DLG sites are unique to Nrf proteins (*NFE2L1* and *NFE2L2*), whereas many other proteins contain an “ETGE motif” and can potentially bind Keap1 [57]. These proteins can potentially displace Nrf2, thus participating in Nrf2 activation [57].

### 3.3. NFκB

**Figure 2:**
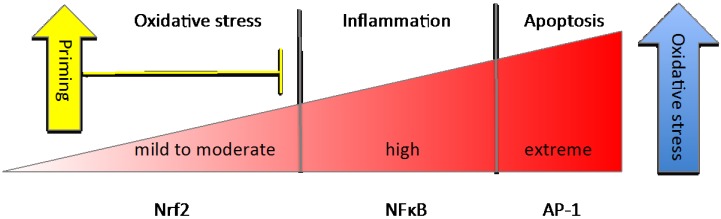
Differential responses to rising oxidative stress.

NFκB (nuclear factor kappa-light-chain-enhancer of activated B cells) is a key transcription factor that regulates cellular immune responses to infection and higher order oxidative stress by coordinating a pro-inflammatory response. Similar to Nrf2, NFκB is sequestered in the cytosol by inhibitor protein IκBα (NFκB inhibitor-alpha). The release of NFκB requires the phosphorylation of IκB by cytosolic protein IKK (IκB kinase); IKKβ is encoded by *IKBKB*. This modification targets IκBα for proteasomal degradation thus releasing NFκB for nuclear translocation. If NFκB-mediated attempts to restore homeostasis fail and oxidative stress rises to extreme levels, AP-1- mediated apoptosis is triggered (Figure 2) [61]. Interestingly, IKKβ contains an ETGE motif [31], therefore it can bind Keap1 and be targeted for ubiquitination [62]. Reducing the IKKβ pool via Keap1 binding reduces IκBα degradation and may be the elusive mechanism by which Nrf2 activation is known to inhibit NFκB activation.

**Figure 3:**
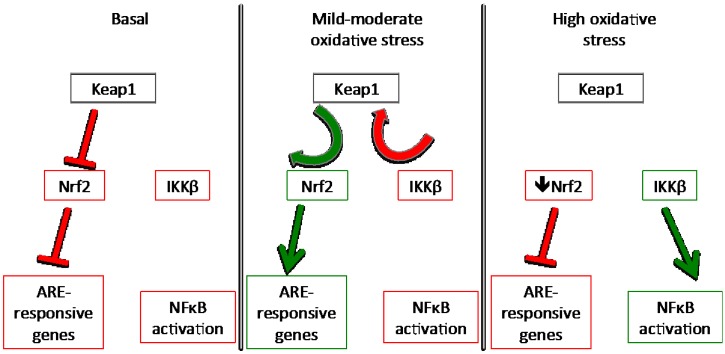
Keap1 as the coordinating factor between Nrf2 activation and NFκB inhibition.

When Nrf2 is released by oxidative triggers, there is an increase in the intracellular pool of unbound Keap1 available to capture more intracellular IKKβ, thus inhibiting the expression of NFκB target genes. Alkylation of Keap1 by electrophilic phytochemicals is reversible; the electrophile is released when the oxidative environment returns to homeostasis and the Nrf2-binding conformation of Keap1 is restored. Beyond a certain threshold in the intracellular oxidation status, Nrf2 can actually promote ROS generation [63]. In true ROS-induced oxidative stress, oxidation of thiol (Cys-SH, 2-) to sulfenic acid (Cys-SOH, 0) is readily reversible, however if sulfenic acid is further oxidized to sulfinic (Cys-SO_2_H, 2+) or sulfonic (Cys-SO_3_H, 4+) acids, the reactions are not reversible, potentially leaving Keap1 unable to revert to a protein-binding conformation [46,64]. This would be expected to abrogate the Keap1 inhibition of IKKβ allowing for an increase in NFκB activation. NFκB has been known to inhibit Nrf2, and so this may be the transition point where oxidative stress becomes inflammation (Figure 3).

## 4. Nutrient Gene Interactions

### 4.1. Phytochemical Activators of Nrf2

#### 4.1.1. Mechanisms of Phytochemical Interaction with Keap1

There are innumerable phytochemicals that have Nrf2 activation abilities and can interact differently with Keap1 sensor thiols. Typically, the potency of an Nrf2 activator is directly related to the speed of its reaction with Keap1 sulfhydryl moieties [43,65]. Alkylating agents are the most potent activators, either displacing the proton of the thiol, or if oxidative conditions rise, thiol is deprotonated to thiolate, which is readily alkylated by electrophilic compounds. Those that reversibly alkylate protein cysteine groups can be released once the oxidative environment becomes reducing again. In a recent high throughput screening study examining the binding affinity of various compounds using an Neh2 reporter assay, all alkylating agents examined were found to be activators [66]. Of the five types of thiol interactions examined, immediate alkylators were the fastest acting, followed with a brief (20 min) lag time by compounds that required simple metabolic modifications to become immediate activators [66].

Most diet-derived phytochemical Nrf2 inducers are Michael acceptors or can be metabolized as such [67]. Michael acceptors are generally defined as acetylene compounds conjugated to an electron-withdrawing group that form reversible alkylating reactions with Keap1 sensor thiols. Curcumin has two carbonyl-conjugated α,β-unsaturated (Michael acceptor) moieties and is a potent activator of Nrf2 as evidenced by its ability to upregulate HO-1 in a rat smooth muscle cell line; however, the saturated form, tetrabutylcurcumin, loses this ability [68]. This suggests that the Michael acceptor moiety is key to Nrf2 activation. Similarly, ginger-derived zerumbone contains a carbonyl-conjugated α,β-unsaturated moiety, and suppresses NFκB activation in cancer cells by reducing the phosphorylation and degradation of IκBα by interfering with IKK activity. This effect is abolished with the loss of the carbonyl group [69]. Zerumbone has also been shown to upregulate the expression of ARE-target genes and downregulate NFκB-targets by direct interaction with Keap1 [70]. The dual ability to both inhibit NFκB activation while activating Nrf2 is also seen in analogs of cinnamate that contain a similar structure: a thioketone-conjugated α,β-unsaturated moiety [71]. When carrot-derived falcarinol is oxidized at C3 to become falcarinone, it has significantly reduced biological activity [72].

#### 4.1.2. Brassica Family Vegetables and Organosulfides

Organosulfides are among the most potent Nrf2 activating phytochemicals. Plants in the Brassica family (broccoli, cabbage, brussel sprouts, turnip, collard greens) are a particularly rich source of organosulfides, primarily glucosinolates and their derivatives. However, different Brassica species present diverse phytochemical profiles. Sulforaphane was the most bioavailable glucosinolate in a broccoli-based diet, while neoglucobrassicin was the main glucosinolate in a pak choi-based diet [73]. Both diets increased NQO1 activity and expression in the colon of azoxymethane (AOM)-treated mice, but only the broccoli diet increased TrxR; however pak choi was more effective at attenuating colonic inflammation than broccoli [73]. Even method of preparation can alter the health effects. Both steamed and cooked broccoli-based diets were protective against ischemic cardiac injury in rats, upregulating Nrf2, SOD1, and SOD2, however only steamed broccoli had any effect on Trx and TrxR expression [74].

The most well studied phytochemical among the glucosinolates is the isothiocyanate, sulforaphane. Nine of the ten current clinical trials evaluating compounds affecting the Nrf2 pathway are focused on sulforaphane [75]. Acute sulforaphane treatment upregulated hepatic GST, GCLC expression and activity along with increased NQO1 mRNA [76]. Longer-term treatment was required to increase NQO1 activity [76]. In rat cardiomyocytes, sulforaphane increased the activation of Nrf2 and increased expression and activity of GSR, GST, TrxR and NQO1 in a PI3K/Akt dependent manner [77]. Mice with induced diabetes received three months of sulforaphane treatment and were either sacrificed immediately or after six months with no further treatment after the initial three-month period. Sulforaphane nearly completely abrogated all signs of oxidative stress and inflammation in the aorta, and this effect persisted strongly even three months after treatment ceased. The authors propose that in addition to the immediate effects on the Nrf2 pathway, the upregulated “program” may be set by epigenetic modifications, such as DNA methylation, conferring a protection that goes beyond transient upregulation of antioxidant enzymes [78]. Interestingly, in a similar study, zinc alone completely blocked inflammation and tissue remodeling associated with T1D in the aorta. Zinc strongly upregulated Nrf2 expression and ARE target NQO1 [79]. In neural tissues, glucoraphanin, a sulforaphane precursor, upregulated Nrf2, and reduced ROS in an induced murine model of Parkinson’s disease, reducing the severity of symptoms [80]. The same compound administered after spinal cord injury protected against cell death, in part by suppressing NFκB activation and subsequent pro-inflammatory cytokine production [81]. Notably, the same pattern appeared in both transcriptomic and proteomic analysis of both sulforaphane-treated, and Keap1 siRNA-treated breast epithelial cells, highlighting that the primary mechanism for the effect of glucosinolates is via the Keap1/Nrf2/ARE pathway [33].

Broccoli seed, rich in various glucosinolates and isothiocyantes, increased the activity of NQO-1 and GST in the stomach, small intestine and liver of wild type, but not Nrf2^−/−^ mice, indicating that Nrf2 is essential for NQO1 induction [82]. GCLC protein was increased in the GI tract of wild type mice only, but not liver; whereas in both normal (RL-34) and cancer (Hepa-1c1c7) murine cell lines, all three (NQO-1, GST, and GCLC) proteins were increased in response to an aqueous extract of broccoli seed in wild type, but not in Nrf2^−^^/−^ cell lines [82]. Mice fed a diet supplemented with Spanish black radish (Brassica) were protected against bone marrow cell death due to the administration of carcinogen DMBA (7,12-dimethylbenz(a)anthracene) [83]. Radish diet upregulated several CYP450 (phase I) enzymes, as well as GST, HO-1, NQO1, and TrxR, resulting in superior detoxification and excretion of the carcinogen, reflected in lower blood levels of DMBA six hours after it was administered [83]. Oral administration of glucobrassicin metabolite, indole-3-carbinol, to female rats protected male fetal rats from bisphenol-A-mediated prostate damage [84]. Sulforaphane has also been shown to protect against alcohol-induced liver steatosis [85] and skin damaged by UV radiation [86], as well as ionizing radiation [87].

#### 4.1.3. Other Fruit and Vegetable-Derived Compounds (Polyphenols, Carotenoids, Polyenes)

Organosulfide compounds are the most extensively studied, however a wide variety of other phytochemical compounds from whole foods interact with Keap1 to similar effect. Whole grape-based diet increased cardiac GSH and GSR activity in hypertensive rats experiencing heart failure [88]. Grape-derived phytochemicals, resveratrol and pterostilbene, were both protective against AOM-induced colon carcinoma by inhibiting NFκB activation and subsequent iNOS and COX-2 expression, concomitant with increased ARE-responsive HO-1 and GSR [89]. Pterostilbene was more effective than resveratrol, particularly upregulating GSR mRNA and HO-1 protein despite a moderate increase in mRNA [89]. Pomegranate protected against induced hepatic carcinogenesis both by Nrf2 upregulation (increased expression of GST, UGT, and NQO1) [90] and NFκB suppression (reduced expression of iNOS, COX-2, and heat shock proteins) [91]. *In vitro*, the methanolic extract of acai berry protected against oxidative stress and lipid peroxidation by increasing the GSH:GSSG ratio and overall glutamate uptake into primary rat astrocytes [92].

Carotenoids are polyenes that lack an electrophilic moiety, however oxidized carotenoid metabolites may have the capacity to upregulate the Nrf2/ARE pathway [93]. Lycopene upregulated Nrf2 resulting in a two-fold increase in NQO1 mRNA and a nearly 2.5-fold increase in GCL mRNA by both lycopene and β-carotene, which corresponded to a two-fold increase in NQO1 protein and a 1.5-fold increase in GCL [94]. Other carotenoids only had marginal effects [94]. Carrot-derived polyacetylene, falcarindiol, forms an S-alkylation adduct with Keap1 at cysteine C151 forming a high molecular weight Keap1, modifying its conformation and releasing Nrf2 [52]. Falcarindiol upregulated catalase, GST, NQO1 and HO-1 in rat hepatocytes and effectively protected against menadione-induced oxidative stress [95]. *In vivo*, falcarindiol acts systemically to upregulate NQO1 and HO-1 in key tissues facing detoxification challenges: liver, kidney, lung, and small intestine [96].

Diethylnitrosamine (DEN) causes hepatocarcinogenesis in rats. Lycopene consumption offered some protection against the severity of carcinogenesis and lipid peroxidation by increasing the production of Nrf2-target enzymes (SOD, catalase, GPx, HO-1) and reducing the phosphorylation of NFκB resulting in the suppression of COX-2 and other targets [97]. Polyphenols, butein and phloretin, upregulated HO-1 and GCLC expression in rat hepatocytes and reduced tert-butyl hydroxyperoxide (t-BHP)-induced ROS production in a GCL and zinc-dependent manner [98]. Cycloartenyl ferulate, a component of rice bran, dose dependently protected HK2 cells against paraquat-mediated ROS production and resulting apoptosis in an Nrf2-dependent manner [99]. Consumption of the methanolic extract of capsicum peppers reduced ROS and inflammatory cell recruitment to lung tissue in a DMBA-induced murine model of asthma [100]. A recent evaluation of the chemopreventive effect of various phytochemicals (chlorophyllin, blueberry, ellagic acid, astaxanthin, and tea polyphenols in order of effectiveness) demonstrated that not only did they upregulate Nrf2 and ARE-target enzymes (GST, NQO1, SOD, catalase, GPx), but also various DNA repair enzymes (OGG1, XPD, XPG, XRCC1) and downregulated specific isoforms of CYP450 responsible for the activation of DMBA [101]. Interestingly, Keap1 expression was dramatically upregulated (400%) [101], which is consistent with the theory that Keap1 is the factor that coordinates the dual Nrf2 activation and NFκB inhibition so consistently observed.

#### 4.1.4. Herbs, Spices and Flavor Enhancers

**Figure 4:**
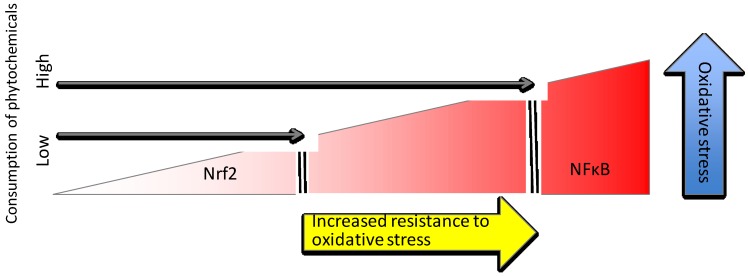
Phytochemical-rich diet increases resistance to oxidative stress and protects against inflammation.

Even herbs, spices and other flavor enhancers used in food preparation can contribute to Nrf2 upregulation. Hydroxytyrosol, a component of olive oil, moderately increased the expression of Nrf2, GCLC and NQO1 in a dose dependent manner and dramatically upregulated HO-1 in human retinal pigment epithelial cells [102]. Notably, hydroxytyrosol increased GSH in untreated cells above control cells that did not receive hydroxytyrosol, and maintained the same elevated GSH levels even after t-BHP treatment to induce oxidative stress [102]. Garlic-derived diallyl sulfide protected rat aortic smooth muscle against TNFα and histamine-induced NFκB activation and ROS production [103]. Compounds isolated from ginger (1-dehydro-6-gingerdione, 6-shogaol and hexahydrocurcumin) all increased NQO1 activity and decreased iNOS activity in LPS stimulated macrophages [104]. Cinnamaldehyde pretreatment of vascular endothelial cells prevented NFκB activation by inhibiting IkBα, resulting in reduced expression of VCAM and ICAM adhesion molecules within three hours TNFα treatment [105]. GSH was also consumed in this process, which lead to an upregulation of Nrf2 target genes, HO-1 and TrxR, as well as a restoration of GSH levels within six hours of TNFα treatment [105]. Carnosic acid, a compound found in herbs, such as rosemary and sage, can cross the blood-brain barrier to support neuronal growth, and upregulates the production of neural protection factors in an Nrf2-dependent manner [106,107]. A tremendous diversity of bioactive phytochemicals is present in most fresh, whole plant-based foods that are capable of upregulating the Keap1/Nrf2/ARE pathway. A diet rich in phytochemicals increases the threshold of oxidative stress that activates the proinflammatory NFκB pathway; the generally enhanced cellular resistance to oxidative stress resulting from the priming of the Keap1/Nrf2/ARE pathway is protective against chronic inflammation (Figure 4).

### 4.2. Trace Minerals

Trace minerals are elements required by the body for normal function in amounts less than approximately 100 mg/day. Trace minerals seem to have a special relationship to non-essential amino acid, cysteine, and its essential precursor, methionine. Zinc and selenium have a special role in redox biology [108].

#### 4.2.1. Zinc

The human body typically contains approximately 2–3 g of zinc and has a daily turnover rate of approximately 20–30 mg [109]. Nearly one third of the global population has insufficient zinc consumption, including nearly 10% of the US population and 13% in Canada [110]. Zinc is highly associated with cysteine. Nearly 97% of zinc-containing metalloproteins in the human genome have at least 1 cysteine in the metal binding site, and more than 40% have four cysteine residues in their metal binding site [111]. Zinc has only one valence state under physiological conditions and so is redox neutral, however it plays a significant role in redox biology [49]. It has been estimated that up to 10% of the human proteome are zinc-binding proteins, whether structural or catalytic [111].

However, protein association is not the only role for zinc in intracellular redox management. It is becoming increasingly clear that “zinc signaling” (fluctuations of zinc concentrations for signal transduction) by free zinc ions (free referring to the rapidly exchanging pool and not ligand-free) may play a role in intracellular signal transduction of the cellular redox state (Table 1). In fact, free zinc may communicate the earliest cell response to changes in the redox state. Under normal homeostatic conditions, plasma zinc is approximately 1 mg/mL. Total intracellular zinc is in the several hundred micromolar range, while free zinc is buffered to the high picomolar to low nanomolar range under normal conditions [112,113]. At picomolar to nanomolar concentrations, free zinc is a potent effector that translates redox signals into zinc signals [21,26].

Triterpenoids are potent Nrf2 activators. Pretreatment with Nrf2 activators upregulates ARE-responsive genes, but zinc treatment alone is sufficient to upregulate Nrf2 itself and many ARE-responsive genes. Microarray analysis of both mouse and rat livers after 4-day pretreatment with oleanolic acid (a triterpenoid) showed a dramatic increase in MT, as well as Nrf2 itself, and other ARE-responsive genes (NQO1, HO-1, GCL, GST, GPx, SOD, catalase) [114]. There was also an increase in hepatic zinc content (30% in mice and slightly in rats) [114]. A similar study administering zinc alone also found a dramatic upregulation of MT in both mice and rat livers [115]. In rat liver, Nrf2 and NQO1 were also upregulated by zinc alone, while HO-1, SOD, and GSH-associated proteins were not [115]. In mouse liver, Nrf2, HO-1, NQO1, and GCLM were upregulated, while SOD other GSH-associated proteins (GST, GPx) were not significantly upregulated [115]. Zinc supplementation provided the same hepatoprotective effects in wild type and MT KO mice, indicating that it is Zn and not MT that has the direct effect on hepatic gene expression [116]. In human colon cancer cells, zinc, but not copper or iron, upregulated HO-1 [117].

Administration of zinc alone to retinal pigment epithelial cells activated the Nrf2-ARE pathway, upregulating *de novo* GSH synthesis (increased GCL mRNA) resulting in a 70% increase in GSH [118]. In a similar cell type, zinc upregulated catalase expression in a dose dependent manner [119]. Zinc depletion reduced catalase activity by 68% and MT by 82% in human fetal retinal pigment epithelial cells, while SOD and GPx were not affected [120]. Zinc deficiency in a glioma (brain cancer) cell line increased oxidative stress and NO production and reduced the DNA binding efficiency of transcription factors p53, AP1 and NFκB without affecting nuclear localization [121]. In rat aortic endothelial cells, NO-mediated protection against H2O2-induced oxidative stress increased GPx and GCL expression with a resulting increase in GSH; these effects were abrogated by the addition of a zinc chelator [20]. In stressed conditions, both free zinc and NO synthesis are required to maintain intracellular GSH; this requires Nrf2 activation but not MTF-1 (a zinc sensing transcription factor) so it is possible the signaling of free zinc is involved [20]. In the brain of rat fetuses from zinc-deficient dams, Nrf2 translocation and GCL mRNA were lower than in controls, and resulted in low levels of GSH [122]. GSH levels remained low despite a dopamine-challenge [122]. The effects were closely mirrored in magnitude in human neuroblastoma IMR-32 cells [122].

#### 4.2.2. Selenium

Both sulfur and selenium are group 16 elements, which are two electrons short of a complete outer shell. Selenium can sometimes displace sulfur in cysteine residues to form selenocysteine. Selenoproteins are key in biological redox systems; selenocysteine (−488 mV) has a higher reduction potential than cysteine (−233 mV) due to its lower pKa, therefore selenol residues a more powerful reducing agents than thiols [9]. Selenocysteine is often referred to as the “twenty first amino acid” because it has no direct genetic code [123]. Selenocysteine is incorporated into proteins by translational recoding where the putative stop codon, UGA, actually codes for selenocysteine when there is an adjacent selenocysteine coding sequence in the mRNA [123]. If there is no selenocysteine available at the time of translation, the peptide is released resulting in a truncated protein. This feature underscores the importance of adequate dietary intake of selenium, since without it important selenoproteins are simply not produced [124].

Selenium intake is a limiting factor in GPx and TrxR synthesis since they both employ selenocysteines in their active sites (SH and SeH), however selenium deficiency also upregulates the expression of other Nrf2 target genes in a compensatory manner [124,125]. AOM-induced tumor number was correlated with selenium intake in a mouse model of colitis; deficient mice developed more numerous and severe tumors than selenium-adequate mice, and the fewest tumors developed in the selenium-supplemented group [126]. Interestingly, sulforaphane pretreatment increased rather than decreased inflammation under selenium deficient conditions, but had an anti-inflammatory effect in selenium adequacy [126]. Sulforaphane and selenium have been shown to synergistically upregulate TrxR in human hepatocytes resulting in better protection against H_2_O_2_-induced apoptosis than either compound alone [127].

## 5. Conclusions

Nrf2 has been recognized as a hormetically regulated pathway in that it reflects a biphasic dose response [50,128]. Electrophilic phytochemicals at low to moderate (dietary) levels induce the activation of Nrf2 with a cell survival-promoting effect, while high (pharmacological) doses have the opposite effect, abrogating Nrf2 and ARE-responsive genes and upregulating NFκB or AP-1 [50,128]. Transient activation of Nrf2 by dietary electrophiles can upregulate antioxidant and chemopreventive enzymes in the absence of actual oxidative stress inducers. Priming the Keap1/Nrf2/ARE pathway by upregulating these enzymes prior to oxidative stress or xenobiotic encounter increases cellular fitness to respond more robustly to oxidative assaults without activating more intense inflammatory NFκB-mediated responses (summarized in Table 1).

**Table 1:** Overview of Nrf2 and changing cellular redox conditions.

**Basal Conditions**
Nrf2 is bound and degraded by Keap1 Nrf2 half-life is about 20 minutes Constitutively active ARE-responsive genes Free zinc is in ~nanoM range
**Priming or Pre-Induction**
No true oxidative stress present Keap1:Nrf2 is actin-bound near plasma membrane; responds to endogenous electrophiles before cytoplasmic reductants have access Level of activation may only reach the release of low affinity DLG “latch”, *de novo* Nrf2 evades Keap1 capture and is available for nuclear translocation *No zinc signals because complete Nrf2 release is limited* Subsequent upregulation of ARE-responsive genes Inducible ARE-responsive genes are activated; GSH-related proteins, HO-1 and NQO1 in particular are most ARE-responsive Transient exposure to dietary electrophilic phytochemicals boosts production of antioxidant and anti-carcinogenic enzymes without triggering an oxidative response with ensuing damage; concomitantly suppresses NFκB Increases the fitness of the antioxidant/chemopreventive response when it truly presents itself, therefore increasing health resilience and reducing the risk of chronic disease development Availability of adequate dietary sulfur, zinc and selenium are required to fully optimize the priming of Keap1/Nrf2/ARE pathway
**Induction**
True oxidative stress present: mild to moderate Keap1 oxidized by wider variety of compounds, including some that may be irreversible *Zinc signals present because of complete release of Nrf2* Depending on level of oxidative stress, *de novo* Nrf2 evades Keap1 capture, Keap1-bound Nrf2 is released leading to further nuclear accumulation of Nrf2 and upregulation of ARE-responsive genes Increased availability of Keap1 to bind IKKβ and suppress the NFκB activation while stress is moderate
**Resolution or Next-Level Response**
Either stress is resolved or Nrf2 can turn pro-oxidative and GSH stores are depleted Higher oxidative state abrogates even Keap1 binding of IKKβ and NFκB suppression comes to an end ARE-responsive genes are suppressed and NFκB targets are upregulated, inducing a pro-inflammatory response to higher order oxidative threat

There is an incredible diversity of electrophilic dietary phytochemicals that interact with the Keap1/Nrf2/ARE pathway. Consuming a wide variety of fresh fruits and vegetables would help to optimize the collective role of Nrf2 regulated proteins: to restore homeostasis from a state of oxidative stress and xenobiotic insult, protecting the integrity of DNA, proteins, membrane and other lipids. Adequate dietary intake of sulfur and trace minerals such as zinc and selenium provide the building blocks necessary to optimize Nrf2-mediated resilience to oxidative stress. Management of the inflammatory and oxidative homeostasis of the body through proper diet may help to slow disease progression or prevent the development of chronic disorders altogether [129,130].

## References

[1] Greene E.R., Huang S., Serhan C.N., Panigrahy D. (2011). Regulation of inflammation in cancer by eicosanoids. Prostaglandin. Other Lipid Mediators.

[2] Khor T.O., Huang M.T., Kwon K.H., Chan J.Y., Reddy B.S., Kong A.N. (2006). Nrf2-Deficient Mice Have an Increased Susceptibility to Dextran Sulfate Sodium-Induced Colitis. Cancer Res..

[3] Kwak M.-K., Wakabayashi N., Itoh K., Motohashi H., Yamamoto M., Kensler T.W. (2002). Modulation of gene expression by cancer chemopreventive dithiolethiones through the Keap1-Nrf2 pathway: Identification of novel gene clusters for cell survival. J. Biol. Chem..

[4] Katsuoka F., Motohashi H., Ishii T., Aburatani H., Engel J.D., Yamamoto M. (2005). Genetic Evidence that Small Maf Proteins Are Essential for the Activation of Antioxidant Response Element-Dependent Genes. Mol. Cell. Biol..

[5] Hayes J.D., McMahon M., Chowdhry S., Dinkova-Kostova A.T. (2010). Cancer chemoprevention mechanisms mediated through the Keap1-Nrf2 pathway. Antioxid. Redox Signal..

[6] Nerland D.E. (2007). The antioxidant/electrophile response element motif. Drug Metab. Rev..

[7] Mieyal J.J., Gallogly M.M., Qanungo S., Sabens E.A., Shelton M.D. (2008). Molecular Mechanisms and Clinical Implications of Reversible Protein *S*-Glutathionylation. Antioxid. Redox Signal..

[8] Shelly C. (2009). Lu Regulation of glutathione synthesis. Mol. Asp. Med..

[9] Jacob C., Giles G.I., Giles N.M., Sies H. (2003). Sulfur and Selenium: The Role of Oxidation State in Protein Structure and Function. Angew. Chem. Int. Ed..

[10] Matés J.M. (2000). Effects of antioxidant enzymes in the molecular control of reactive oxygen species toxicology. Toxicology.

[11] Dinkova-Kostova A.T., Talalay P. (2010). NAD(P)H:quinone acceptor oxidoreductase 1 (NQO1), a multifunctional antioxidant enzyme and exceptionally versatile cytoprotector. Arch. Biochem. Biophys..

[12] Zhang D.D., Hannink M. (2003). Distinct Cysteine Residues in Keap1 Are Required for Keap1-Dependent Ubiquitination of Nrf2 and for Stabilization of Nrf2 by Chemopreventive Agents and Oxidative Stress. Mol. Cell. Biol..

[13] Mustacich D., Powis G. (2000). Thioredoxin reductase. Biochem. J..

[14] Coyle P., Philcox J.C., Carey L.C., Rofe A.M. (2002). Metallothionein: The multipurpose protein. Cell. Mol. Life Sci..

[15] Krȩżel A., Maret W. (2007). Dual Nanomolar and Picomolar Zn(II) Binding Properties of Metallothionein. J. Am. Chem. Soc..

[16] Ohtsuji M., Katsuoka F., Kobayashi A., Aburatani H., Hayes J.D., Yamamoto M. (2008). Nrf1 and Nrf2 Play Distinct Roles in Activation of Antioxidant Response Element-dependent Genes. J. Biol. Chem..

[17] Alam J., Stewart D., Touchard C., Boinapally S., Choi A.M.K., Cook J.L. (1999). Nrf2, a Cap‘n’Collar Transcription Factor, Regulates Induction of the Heme Oxygenase-1 Gene. J. Biol. Chem..

[18] Kröncke K.D., Fehsel K., Schmidt T., Zenke F.T., Dasting I., Wesener J.R., Betterman H., Breunig K.D., Kolb-Bachofen V. (1994). Nitric Oxide Destroys Zinc-Sulfur Clusters Inducing Zinc Release from Metallothionein and Inhibition of the Zinc Finger-Type Yeast Transcription Activator LAC9. Biochem. Biophys. Res. Commun..

[19] Maret W. (1994). Oxidative metal release from metallothionein via zinc-thiol/disulfide interchange. Proc. Natl. Acad. Sci. USA.

[20] Cortese-Krott M.M., Suschek C.V., Wetzel W., Kroncke K.D., Kolb-Bachofen V. (2009). Nitric oxide-mediated protection of endothelial cells from hydrogen peroxide is mediated by intracellular zinc and glutathione. AJP Cell Physiol..

[21] Maret W. (2011). Redox biochemistry of mammalian metallothioneins. J. Biol. Inorg. Chem..

[22] Krȩżel A., Maret W. (2007). Thionein/metallothionein control Zn(II) availability and the activity of enzymes. J. Biol. Inorg. Chem..

[23] Hasse H., Rink L. (2013). Zinc signals and immune function. BioFactors.

[24] Maret W., Vallee B.L. (1998). Thiolate ligands in metallothionein confer redox activity on zinc clusters. Proc. Natl. Acad. Sci. USA.

[25] Jacob C., Maret W., Vallee B.L. (1999). Selenium redox biochemistry of zinc—Sulfur coordination sites in proteins and enzymes. Proc. Natl. Acad. Sci. USA.

[26] Maret W., Jacob C., Vallee B.L., Fischer E. (1999). Inhibitory sites in enzymes: Zinc removal and reactivation by thionein. Proc. Natl. Acad. Sci. USA.

[27] Talalay P., Dinkova-Kostova A.T., Holtzclaw W.D. (2003). Importance of phase 2 gene regulation in protection against electrophile and reactive oxygen toxicity and carcinogenesis. Adv. Enzym. Regul..

[28] Kaminsky L.S., Zhang Q.-Y. (2003). The small intestine as a xenobiotic-metabolizing organ. Drug Metab. Dispos..

[29] Sturgill M.G., Lambert G.H. (1997). Xenobiotic-induced hepatoxicity: Mechanisms of liver injury and methods of monitoring hepatic function. Clin. Chem..

[30] Yang Y.M., Noh K., Han C.Y., Kim S.G. (2010). Transactivation of Genes Encoding for Phase II Enzymes and Phase III Transporters by Phytochemical Antioxidants. Molecules.

[31] Kim J.-E., You D.-J., Lee C., Ahn C., Seong J.Y., Hwang J.-I. (2010). Suppression of NF-kappaB signaling by Keap1 regulation of IKK-beta activity through autophagic degradation and inhibition of phosphorylation. Cell Signal..

[32] Ashford J.H.M.J., Ashford M.L.J. (2012). Nrf2 Orchestrates Fuel Partitioning for Cell Proliferation. Cell Metab..

[33] Agyeman A.S., Chaerkady R., Shaw P.G., Davidson N.E., Visvanathan K., Pandey A., Kensler T.W. (2011). Transcriptomic and proteomic profiling of KEAP1 disrupted and sulforaphane-treated human breast epithelial cells reveals common expression profiles. Breast Cancer Res. Treat.

[34] Mitsuishi Y., Taguchi K., Kawatani Y., Shibata T., Nukiwa T., Aburatani H., Yamamoto M., Motohashi H. (2012). Nrf2 Redirects Glucose and Glutamine into Anabolic Pathways in Metabolic Reprogramming. Cancer Cell.

[35] Wu K.C., Cui J.Y., Klaassen C.D. (2011). Beneficial Role of Nrf2 in Regulating NADPH Generation and Consumption. Toxicol. Sci..

[36] Kang M.-I., Kobayashi A., Wakabayashi N., Kim S.G., Yamamoto M. (2004). Scaffolding of Keap1 to the actin cytoskeleton controls the function of Nrf2 as key regulator ofcytoprotective phase 2 genes. Proc. Natl. Acad. Sci. USA.

[37] Adams J., Kelso R., Cooley L. (1999). The kelch repeat superfamily of proteins: Propellers of cell function. Trends Cell Biol..

[38] Dinkova-Kostova A.T., Holtzclaw W.D., Wakabayashi N. (2005). Keap1, the Sensor for Electrophiles and Oxidants that Regulates the Phase 2 Response, Is a Zinc Metalloprotein. Biochemistry.

[39] Velichkova M., Hasson T. (2003). Keap1 in Adhesion Complexes. Cell Motil. Cytoskelet..

[40] Cullinan S.B., Gordan J.D., Jin J., Harper J.W., Diehl J.A. (2004). The Keap1-BTB Protein Is an Adaptor That Bridges Nrf2 to a Cul3-Based E3 Ligase: Oxidative Stress Sensing by a Cul3-Keap1 Ligase. Mol. Cell. Biol..

[41] Kobayashi A., Kang M.I., Okawa H., Ohtsuji M., Zenke Y., Chiba T., Igarashi K., Yamamoto M. (2004). Oxidative Stress Sensor Keap1 Functions as an Adaptor for Cul3-Based E3 Ligase To Regulate Proteasomal Degradation of Nrf2. Mol. Cell. Biol..

[42] Zipper L.M., Mulcahy R.T. (2002). The Keap1 BTB/POZ Dimerization Function Is Required to Sequester Nrf2 in Cytoplasm. J. Biol. Chem..

[43] Dinkova-Kostova A.T., Holtzclaw W.D., Cole R.N., Itoh K., Wakabayashi N., Katoh Y., Yamamoto M., Talalay P. (2002). Direct evidence that sulfhydryl groups of Keap1 are the sensors regulating induction of phase 2 enzymes that protect against carcinogens and oxidants. Proc. Natl. Acad. Sci. USA.

[44] Tong K.I., Kobayashi A., Katsuoka F., Yamamoto M. (2006). Two-site substrate recognition model for the Keap1-Nrf2 system: A hinge and latch mechanism. Biol. Chem..

[45] Ogura T., Tong K.I., Mio K., Maruyama Y., Kurokawa H., Sato C., Yamamoto M. (2010). Keap1 is a forked-stem dimer structure with two large spheres enclosing the intervening, double glycine repeat, and *C*-terminal domains. Proc. Natl. Acad. Sci. USA.

[46] Forman H.J., Ursini F., Maiorino M. (2014). An overview of mechanisms of redox signaling. J. Mol. Cell. Cardiol..

[47] Wakabayashi N., Dinkova-Kostova A.T., Holtzclaw W.D., Kang M.-I., Kobayashi A., Yamamoto M., Kensler T.W., Talalay P. (2004). Protection against electrophile and oxidant stress by induction of the phase 2 response: Fate of cysteines of the Keap1 sensor modified by inducers. Proc. Natl. Acad. Sci. USA.

[48] Powell S.R. (2000). The antioxidant properties of zinc. J. Nutr..

[49] Eide D.J. (2011). The oxidative stress of zinc deficiency. Metallomics.

[50] Mattson M.P., Cheng A. (2006). Neurohormetic phytochemicals: Low-dose toxins that induce adaptive neuronal stress responses. Trends Neurosci..

[51] Hong F., Sekhar K.R., Freeman M.L., Liebler D.C. (2005). Specific Patterns of Electrophile Adduction Trigger Keap1 Ubiquitination and Nrf2 Activation. J. Biol. Chem..

[52] Ohnuma T., Nakayama S., Ana E., Nishiyama T., Ogura K., Hiratsuka A. (2010). Activation of the Nrf2/ARE pathway via S-alkylation of cysteine 151 in the chemopreventive agent-sensor Keap1 protein by falcarindiol, a conjugated diacetylene compound. Toxicol. Appl. Pharmacol..

[53] Luo Y., Eggler A.L., Liu D., Liu G., Mesecar A.D., van Breemen R.B. (2007). Sites of alkylation of human Keap1 by natural chemoprevention agents. J. Am. Soc. Mass Spectrom..

[54] Eggler A.L., Liu G., Pezzuto J.M., van Breemen R.B., Mesecar A.D. (2005). Modifying specific cysteines of the electrophile-sensing human Keap1 protein is insufficient to disrupt binding to the Nrf2 domain Neh2. Proc. Natl. Acad. Sci. USA.

[55] Itoh K., Chiba T., Takahashi S., Ishii T., Igarashi K., Katoh Y., Oyake T., Hayashi N., Satoh K., Hatayama I. (1997). An Nrf2/Small Maf Heterodimer Mediates the Induction of Phase II Detoxifying Enzyme Genes through Antioxidant Response Elements. Biochem. Biophys. Res. Commun..

[56] Tong K.I., Padmanabhan B., Kobayashi A., Shang C., Hirotsu Y., Yokoyama S., Yamamoto M. (2007). Different Electrostatic Potentials Define ETGE and DLG Motifs as Hinge and Latch in Oxidative Stress Response. Mol. Cell. Biol..

[57] Hast B.E., Goldfarb D., Mulvaney K.M., Hast M.A., Siesser P.F., Yan F., Hayes D.N., Major M.B. (2013). Proteomic Analysis of Ubiquitin Ligase KEAP1 Reveals Associated Proteins That Inhibit NRF2 Ubiquitination. Cancer Res..

[58] Tong K.I., Katoh Y., Kusunoki H., Itoh K., Tanaka T., Yamamoto M. (2006). Keap1 Recruits Neh2 through Binding to ETGE and DLG Motifs: Characterization of the Two-Site Molecular Recognition Model. Mol. Cell. Biol..

[59] Kobayashi A., Kang M.I., Watai Y., Tong K.I., Shibata T., Uchida K., Yamamoto M. (2005). Oxidative and Electrophilic Stresses Activate Nrf2 through Inhibition of Ubiquitination Activity of Keap1. Mol. Cell. Biol..

[60] Watai Y., Kobayashi A., Nagase H., Mizukami M., McEvoy J., Singer J.D., Itoh K., Yamamoto M. (2007). Subcellular localization and cytoplasmic complex status of endogenous Keap1. Genes Cell..

[61] Woods C.G., Fu J., Xue P., Hou Y., Pluta L.J., Yang L., Zhang Q., Thomas R.S., Andersen M.E., Pi J. (2009). Dose-dependent transitions in Nrf2-mediated adaptive response and related stress responses to hypochlorous acid in mouse macrophages. Toxicol. Appl. Pharmacol..

[62] Lee D.-F., Kuo H.-P., Liu M., Chou C.-K., Xia W., Du Y., Shen J., Chen C.-T., Huo L., Hsu M.-C. (2009). Keap1 E3 ligase-mediated downregulation of NFkB signaling by targeting IKK-beta. Mol. Cell.

[63] Zucker S.N., Fink E.E., Bagati A., Mannava S., Bianchi-Smiraglia A., Bogner P.N., Wawrzyniak J.A., Foley C., Leonova K.I., Grimm M.J. (2014). Nrf2 Amplifies Oxidative Stress via Induction of Klf9. Mol. Cell.

[64] Poole L.B., Karplus P.A., Claiborne A. (2004). Protein sulfenic acids in redox signaling. Annu. Rev. Pharmacol. Toxicol..

[65] Dinkova-Kostova A.T., Massiah M.A., Bozak R.E., Hicks R.J., Talalay P. (2001). Potency of Michael reaction acceptors as inducers of enzymes that protect against carcinogenesis depends on their reactivity with sulfhydryl groups. Proc. Natl. Acad. Sci. USA.

[66] Smirnova N.A., Haskew-Layton R.E., Basso M., Hushpulian D.M., Payappilly J.B., Speer R.E., Ahn Y.-H., Rakhman I., Cole P.A., Pinto J.T., Ratan R.R. (2011). Development of Neh2-Luciferase Reporter and Its Application for High Throughput Screening and Real-Time Monitoring of Nrf2 Activators. Chem. Biol..

[67] Dinkova-Kostova A.T., Holtzclaw W.D., Kensler T.W. (2005). The Role of Keap1 in Cellular Protective Responses. Chem. Res. Toxicol..

[68] Pae H.-O., Jeong G.-S., Jeong S.-O., Kim H.S., Kim S.-A., Kim Y.-C., Yoo S.-J., Kin H.-D., Chung H.-T. (2007). Roles of heme oxygenase-1 in curcumin-induced growth inhibition in rat smooth muscle cells. Exp. Mol. Med..

[69] Takada Y., Murakami A., Aggarwal B.B. (2005). Zerumbone abolishes NF-κB and IκBα kinase activation leading to suppression of antiapoptotic and metastatic gene expression, upregulation of apoptosis, and downregulation of invasion. Oncogene.

[70] Ohnishi K., Irie K., Murakami A. (2009). *In vitro* covalent binding of zerumbone, a chemopreventive food factor. Biosci. Biotechnol. Biochem..

[71] Kumar S., Singh B.K., Prasad A.K., Parmar V.S., Biswal S., Ghosh B. (2013). Ethyl 3’,4’,5’-trimethoxythionocinnamate modulates NF-κB and Nrf2 transcription factors. Eur. J. Pharmacol..

[72] Purup S., Larsen E., Christensen L.P. (2009). Differential Effects of Falcarinol and Related Aliphatic C 17-Polyacetylenes on Intestinal Cell Proliferation. J. Agric. Food Chem..

[73] Lippmann D., Lehmann C., Florian S., Barknowitz G., Haack M., Mewis I., Wiesner M., Schreiner M., Glatt H., Brigelius-Flohé R. (2014). Glucosinolates from pak choi and broccoli induce enzymes and inhibit inflammation and colon cancer differently. Food Funct..

[74] Mukherjee S., Lekli I., Ray D., Gangopadhyay H., Raychaudhuri U., Das D.K. (2010). Comparison of the protective effects of steamed and cooked broccolis on ischaemia—Reperfusion-induced cardiac injury. Br. J. Nutr..

[75] U.S. National Institutes of Health ClinicalTrials.gov. http://clinicaltrials.gov.

[76] Philbrook N.A., Winn L.M. (2014). Sub-chronic sulforaphane exposure in CD-1 pregnant mice enhances maternal NADPH quinone oxidoreductase 1 (NQO1) activity and mRNA expression of NQO1, glutathione *S*-transferase, and glutamate-cysteine ligase. Reprod. Toxicol..

[77] Leoncini E., Malaguti M., Angeloni C., Motori E., Fabbri D., Hrelia S. (2011). Cruciferous Vegetable Phytochemical Sulforaphane Affects Phase II Enzyme Expression and Activity in Rat Cardiomyocytes through Modulation of Akt Signaling Pathway. J. Food Sci..

[78] Miao X., Bai Y., Sun W., Cui W., Xin Y., Wang Y., Tan Y., Miao L., Fu Y., Su G., Cai L. (2012). Sulforaphane prevention of diabetes-induced aortic damage was associated with the up-regulation of Nrf2 and its down-stream antioxidants. Nutr. Metab..

[79] Miao X., Wang Y., Sun J., Sun W., Tan Y., Cai L., Zheng Y., Su G., Liu Q., Wang Y. (2013). Zinc protects against diabetes-induced pathogenic changes in the aorta: Roles of metallothionein and nuclear factor (erythroid-derived 2)-like 2. Cardiovasc. Diabetol..

[80] Galuppo M., Iori R., de Nicola G.R., Bramanti P., Mazzon E. (2013). Anti-inflammatory and anti-apoptotic effects of (RS)-glucoraphanin bioactivated with myrosinase in murine sub-acute and acute MPTP-induced Parkinson’s disease. Bioorganic Med. Chem..

[81] Galuppo M., Giacoppo S., de Nicola G.R., Iori R., Mazzon E., Bramanti P. (2013). Journal of the Neurological Sciences. J. Neurol. Sci..

[82] McWalter G.K., Higgins L.G., McLellan L.I., Henderson C.J., Song L., Thornalley P.J., Itoh K., Yamamoto M., Hayes J.D. (2004). Transcription Factor Nrf2 Is Essential for Induction of NAD(P)H:Quinone Oxidoreductase 1, Glutathione *S-*Transferases, and Glutamate Cysteine Ligase by Broccoli Seeds and Isothiocyanates. J. Nutr..

[83] N’jai A.U., Kemp M.Q., Metzger B.T., Hanlon P.R., Robbins M., Czuyprynski C., Barnes D.M. (2012). Spanish Black Radish (*Raphanus Sativus* L. Var. niger) Diet Enhances Clearance of DMBA and Diminishes Toxic Effects on Bone Marrow Progenitor Cells. Nutr. Cancer.

[84] Brandt J.Z., Silveira L.T.R., Grassi T.F., Anselmo-Franci J.A., Fávaro W.J., Felisbino S.L., Barbisan L.F., Scarano W.R. (2014). Indole-3-carbinol attenuates the deleterious gestational effects of bisphenol A exposure on the prostate gland of male F1 rats. Reprod. Toxicol..

[85] Zhou R., Lin J., Wu D. (2014). Sulforaphane induces Nrf2 and protects against CYP2E1-dependent binge alcohol-induced liver steatosis. Biochim. Biophys. Acta.

[86] Kleszczyński K., Ernst I.M.A., Wagner A.E., Kruse N., Zillikens D., Rimbach G., Fischer T.W. (2013). Sulforaphane and phenylethyl isothiocyanate protect human skin against UVR-induced oxidative stress and apoptosis: Role of Nrf2-dependent gene expression and antioxidant enzymes. Pharmacol. Res..

[87] Mathew S.T., Bergström P., Hammarsten O. (2014). Repeated Nrf2 stimulation using sulforaphane protects fibroblasts from ionizing radiation. Toxicol. Appl. Pharmacol..

[88] Seymour E.M., Bennink M.R., Bolling S.F. (2013). Diet-relevant phytochemical intake affects the cardiac AhR and nrf2 transcriptome and reduces heart failure in hypertensive rats. J. Nutr. Biochem..

[89] Chiou Y.-S., Tsai M.-L., Nagabhushanam K., Wang Y.-J., Wu C.-H., Ho C.-T., Pan M.-H. (2011). Pterostilbene Is More Potent than Resveratrol in Preventing Azoxymethane (AOM)-Induced Colon Tumorigenesis via Activation of the NF-E2-Related Factor 2 (Nrf2)-Mediated Antioxidant Signaling Pathway. J. Agricul. Food Chem..

[90] Bishayee A., Bhatia D., Thoppil R.J., Darvesh A.S., Nevo E., Lansky E.P. (2011). Pomegranate-mediated chemoprevention of experimental hepatocarcinogenesis involves Nrf2-regulated antioxidant mechanisms. Carcinogenesis.

[91] Bishayee A., Thoppil R.J., Darvesh A.S., Ohanyan V., Meszaros J.G., Bhatia D. (2013). Pomegranate phytoconstituents blunt the inflammatory cascade in a chemically induced rodent model of hepatocellular carcinogenesis. J. Nutr. Biochem..

[92] Da Silva Santos V., Bisen-Hersh E., Yu Y., Cabral I.S., Nardini V., Culbreth M., Teixeira da Rocha J.B., Barbosa F., Aschner M. (2014). Anthocyanin-Rich Açaí (*Euterpe oleracea* Mart.) Extract Attenuates Manganese-Induced Oxidative Stress in Rat Primary Astrocyte Cultures. J. Toxicol. Environ. Health Part A.

[93] Linnewiel K., Ernst H., Caris-Veyrat C., Ben-Dor A., Kampf A., Salman H., Danilenko M., Levy J., Sharoni Y. (2009). Structure activity relationship of carotenoid derivatives in activation of the electrophile/antioxidant response element transcription system. Free Radic. Biol. Med..

[94] Ben-Dor A., Steiner M., Gheber L., Danilenko M., Dubi N., Linnewiel K., Zick A., Sharoni Y., Levy J. (2005). Carotenoids activate the antioxidant response element transcription system. Mol. Cancer Ther..

[95] Ohnuma T., Komatsu T., Nakayama S., Nishiyama T., Ogura K., Hiratsuka A. (2009). Induction of antioxidant and phase 2 drug-metabolizing enzymes by falcarindiol isolated from *Notopterygium incisum* extract, which activates the Nrf2/ARE pathway, leads to cytoprotection against oxidative and electrophilic stress. Arch. Biochem. Biophys..

[96] Ohnuma T., Ana E., Hoashi R., Takeda Y., Nishiyama T., Ogura K., Hiratsuka A. (2011). Dietary diacetylene falcarindiol induces phase 2 drug-metabolizing enzymes and blocks carbon tetrachloride-induced hepatotoxicity in mice through suppression of lipid peroxidation. Biol. Pharm. Bull..

[97] Sahin K., Orhan C., Tuzcu M., Sahin N., Ali S., Bahcecioglu I.H., Guler O., Ozercan I., Ilhan N., Kucuk O. (2014). Orally Administered Lycopene Attenuates Diethylnitrosamine-Induced Hepatocarcinogenesis in Rats by Modulating Nrf-2/HO-1 and Akt/mTOR Pathways. Nutr. Cancer.

[98] Yang Y.-C., Lii C.-K., Lin A.-H., Yeh Y.-W., Yao H.-T., Li C.-C., Liu K.-L., Chen H.-W. (2011). Induction of glutathione synthesis and heme oxygenase 1 by the flavonoids butein and phloretin is mediated through the ERK/Nrf2 pathway and protects against oxidative stress. Free Rad. Biol. Med..

[99] Hong G.-L., Liu J.-M., Zhao G.-J., Wang L., Liang G., Wu B., Li M.-F., Qiu Q.-M., Lu Z.-Q. (2013). The reversal of paraquat-induced mitochondria- mediated apoptosis by cycloartenyl ferulate, the important role of Nrf2 pathway. Exp. Cell Res..

[100] Jang H.-Y., Kim S.-M., Yuk J.-E., Kwon O.-K., Oh S.-R., Lee H.-K., Jeong H., Ahn K.-S. (2011). *Capsicum annuum* L. Methanolic Extract Inhibits Ovalbumin-Induced Airway Inflammation and Oxidative Stress in a Mouse Model of Asthma. J. Med. Food.

[101] Kavitha K., Thiyagarajan P., Nandhini J.R., Mishra R., Nagini S. (2013). Chemopreventive effects of diverse dietary phytochemicals against DMBA-induced hamster buccal pouch carcinogenesis via the induction of Nrf2-mediated cytoprotective antioxidant, detoxification, and DNA repair enzymes. Biochimie.

[102] Zou X., Feng Z., Li Y., Wang Y., Wertz K., Weber P., Fu Y., Liu J. (2012). Stimulation of GSH synthesis to prevent oxidative stress-induced apoptosis by hydroxytyrosol in human retinal pigment epithelial cells: Activation of Nrf2 and JNK-p62/SQSTM1 pathways. J. Nutr. Biochem..

[103] Ho C.-Y., Weng C.-J., Jhang J.-J., Cheng Y.-T., Huang S.-M., Yen G.-C. (2014). Diallyl sulfide as a potential dietary agent to reduce TNF-α- and histamine-induced proinflammatory responses in A7r5 cells. Mol. Nutr. Food Res..

[104] Li F., Wang Y., Parkin K.L., Nitteranon V., Liang J., Yang W., Li Y., Zhang G., Hu Q. (2011). Isolation of quinone reductase (QR) inducing agents from ginger rhizome and their *in vitro* anti-inflammatory activity. Food Res. Int..

[105] Liao B.-C., Hsieh C.-W., Liu Y.-C., Tzeng T.-T., Sun Y.-W., Wung B.-S. (2008). Cinnamaldehyde inhibits the tumor necrosis factor-α-induced expression of cell adhesion molecules in endothelial cells by suppressing NF-κB activation: Effects upon IκB and Nrf2. Toxicol. Appl. Pharmacol..

[106] Kosaka K., Mimura J., Itoh K., Satoh T., Shimojo Y., Kitajima C., Maruyama A., Yamamoto M., Shirasawa T. (2010). Role of Nrf2 and p62/ZIP in the neurite outgrowth by carnosic acid in PC12h cells. J. Biochem..

[107] Mimura J., Kosaka K., Maruyama A., Satoh T., Harada N., Yoshida H., Satoh K., Yamamoto M., Itoh K. (2011). Nrf2 regulates NGF mRNA induction by carnosic acid in T98G glioblastoma cells and normal human astrocytes. J. Biochem..

[108] Klotz L.-O., Kröncke K.D., Buchczyk D.P., Sies H. (2003). Role of copper, zinc, selenium and tellurium in the cellular defense against oxidative and nitrosative stress. J. Nutr..

[109] Maret W., Sandstead H.H. (2006). Zinc requirements and the risks and benefits of zinc supplementation. J. Trace Elem. Med. Biol..

[110] Bhutta Z., Brown K.H., Gibson R.S., Hotz C., King J.C., Lönnerdal B., Lopez de Romaña Forga D., Peerson J.M., Rivera J.A., Ruel M.T. (2004). Estimated Risk of Zinc Deficiency by Country. Food Nutr. Bull..

[111] Andreini C., Banci L., Bertini I., Rosato A. (2006). Counting the zinc-proteins encoded in the human genome. J. Proteome Res..

[112] Bozym R.A., Thompson R.B., Stoddard A.K., Fierke C.A. (2006). Measuring Picomolar Intracellular Exchangeable Zinc in PC-12 Cells Using a Ratiometric Fluorescence Biosensor. ACS Chem. Biol..

[113] Maret W. (2011). Metals on the move: Zinc ions in cellular regulation and in the coordination dynamics of zinc proteins. Biometals.

[114] Liu J., Wu Q., Lu Y.-F., Pi J. (2008). New insights into generalized hepatoprotective effects of oleanolic acid: Key roles of metallothionein and Nrf2 induction. Biochem. Pharmacol..

[115] Liu J., Zhou Z.-X., Zhang W., Bell M.W., Waalkes M.P. (2009). Changes in hepatic gene expression in response to hepatoprotective levels of zinc. Liver Int..

[116] Zhou Z.-X., Sun X., Lambert J.C., Saari J.T., Kang Y.J. (2002). Metallothionein-Independent Zinc Protection from Alcoholic Liver Injury. Am. J. Pathol..

[117] Smith A.F., Loo G. (2012). Upregulation of haeme oxygenase-1 by zinc in HCT-116 cells. Free Radic. Res..

[118] Ha K.-N., Chen Y., Cai J., Sternberg P. (2006). Increased Glutathione Synthesis throughan ARE-Nrf2-Dependent Pathway by Zinc in the RPE: Implication for Protection against Oxidative Stress. Investig. Ophthamol. Vis. Sci..

[119] Tate D.J., Miceli M.V., Newsome D.A. (1997). Zinc induces catalase expression in cultured fetal human retinal pigment epithelial cells. Curr. Eye Res..

[120] Tate D.J., Miceli M.V., Newsome D.A., Alcock N.W., Oliver P.D. (1995). Influence of zinc on selected cellular functions of cultured human retinal pigment epithelium. Curr. Eye Res..

[121] Ho E., Ames B.N. (2002). Low intracellular zinc induces oxidative DNA damage, disrupts p53, NFkB, and AP1 DNA binding, and affects DNA repair in a rat glioma cell line. Proc. Natl. Acad. Sci. USA.

[122] Omata Y., Salvador G.A., Supasai S., Keenan A.H., Oteiza P.I. (2013). Decreased Zinc Availability Affects Glutathione Metabolism in Neuronal Cells and in the Developing Brain. Toxicol. Sci..

[123] Bröcker M.J., Ho J.M.L., Church G.M., Söll D., O’Donoghue P. (2013). Recoding the Genetic Code with Selenocysteine. Angew. Chem. Int. Ed..

[124] Müller M., Banning A., Brigelius-Flohé R., Kipp A. (2010). Nrf2 target genes are induced under marginal selenium-deficiency. Genes Nutr..

[125] Brigelius-Flohé R., Müller M., Lippmann D., Kipp A.P. (2012). The Yin and Yang of Nrf2-Regulated Selenoproteins in Carcinogenesis. Int. J. Cell Biol..

[126] Krehl S., Loewinger M., Florian S., Kipp A.P., Banning A., Wessjohann L.A., Brauer M.N., Iori R., Esworthy R.S., Chu F.F. (2012). Glutathione peroxidase-2 and selenium decreased inflammation and tumors in a mouse model of inflammation-associated carcinogenesis whereas sulforaphane effects differed with selenium supply. Carcinogenesis.

[127] Li D., Wang W., Shan Y., Barrera L.N., Howie A.F., Beckett G.J., Wu K., Bao Y. (2012). Synergy between sulforaphane and selenium in the up-regulation of thioredoxin reductase and protection against hydrogen peroxide-induced cell death in human hepatocytes. Food Chem..

[128] Mattson M.P. (2008). Hormesis defined. Ageing Res. Rev..

[129] Gillies P.J. (2007). Preemptive nutrition of pro-inflammatory states: A nutrigenomic model. Nutr. Rev..

[130] Szarc vel Szic K., Ndlovu M.N., Haegeman G., Vanden Berghe W. (2010). Nature or nurture: Let food be your epigenetic medicine in chronic inflammatory disorders. Biochem. Pharmacol..

